# Risk-Factor Profile and Comorbidities in 2398 Patients With Newly Diagnosed Hypertension From the Abuja Heart Study

**DOI:** 10.1097/MD.0000000000001660

**Published:** 2015-10-02

**Authors:** Dike B. Ojji, Elena Libhaber, John J. Atherton, Bolaji Abdullahi, Ada Nwankwo, Karen Sliwa

**Affiliations:** From the Cardiology Unit (DBO, BA, AN), Department of Medicine, Faculty of Health Sciences, College of Medicine, University of Abuja Teaching Hospital, Gwagwalada, Abuja, Nigeria; Soweto Cardiovascular Research Unit (DBO, EL, KS), School of Clinical Medicine, University of Witwatersrand, South Africa; Department of Cardiology (JJA), Royal Brisbane and Women's Hospital and University of Queensland School of Medicine, Brisbane, Australia; and Hatter Institute for Cardiovascular Research in Africa (KS), Department of Medicine, Faculty of Health Sciences, University of Cape Town, South Africa.

## Abstract

Risk factors, comorbidities, and end-organ damage in newly diagnosed hypertension (HT) are poorly described in larger cohorts of urban African patients undergoing epidemiological transition. We therefore decided to characterize a large cohort of hypertensive subjects presenting to a tertiary health center in sub-Saharan Africa.

It is an observational cross-sectional study. We prospectively collected detailed clinical, biochemical, electrocardiography, and echocardiography data of all subjects with HT as the primary diagnosis in patients presenting at the Cardiology Unit of the University of Abuja Teaching Hospital over an 8-year period.

Of 2398 subjects, 1187 patients (49.4%) were female with a mean age of 51 ± 12.8 years. Presenting symptoms and signs were most commonly palpitation in 691 (28.8%) followed by dyspnoea on exertion in 541 (22.6%), orthopnea in 532 (22.2%), pedal oedema in 468 (19.5%), paroxysmal nocturnal dyspnoea in 332 (13.8%), whereas only 31 (1.3%) presented with chest pain. Risk factors were obesity in 671 (28%); 523 (21.8%) had total cholesterol >5.2 mmol/L, diabetes mellitus was present in 201 (8.4%) and 187 (7.8%) were smokers.

End-organ damage was present in form of echocardiographic left ventricular hypertrophy in 1336 (55.7%) followed by heart failure in 542 (22.6%). Arrhythmias occurred in 110 (4.6%) of cases, cerebrovascular accident in 103 (4.3%), chronic kidney disease in 26 (1.1%), hypertensive encephalopathy in 10 (0.4%), and coronary artery disease in 6 (0.26%). There were marked differences in sex as women were more obese and men presented with more advanced disease.

The burden of HT and its complications in this carefully characterized African cohort is quite enormous with more than three-fourth having one form of complication. The need of effective primary and secondary preventive measures to be mapped out to tackle this problem cannot be overemphasized.

## INTRODUCTION

Hypertension (HT) constitutes a major health concern worldwide.^1^ According to recent estimates, the worldwide prevalence of HT in 2000 was approximately 26%, totaling about 1 billion people,^2^ and along with the aging population, HT prevalence has been projected to increase to ≥29% by 2025.^2^ In a recent systematic review, the overall prevalence of HT in sub-Saharan Africa was estimated at 16.2%, with an estimated number of hypertensive individuals to be 74.7 million.^3^ In Nigeria, the crude prevalence of HT has been estimated to be 20% to 25%.^4^

Even though HT affects every ethnic group, the consequences are said to be more devastating among blacks and include congestive cardiac failure, chronic kidney disease, cerebrovascular disease, cardiovascular mortality, and sudden cardiac death.^5^ In the Heart of Soweto Study, HT was diagnosed in 52% of Black Africans and was often associated with advanced forms of heart disease.^6^ It was also found in the Heart of Soweto study that there were some sex differences in presentation and complication profile of the hypertensive cohort.^6^ Despite the high prevalence of HT and its complications in sub-Saharan Africa, there are still a paucity of large sample data describing the pattern of presentation of HT and its complications. This is required to define the burden of disease imposed by HT, which will inform health policy and resource allocation. We therefore decided to study the pattern of presentation of HT and its complications in large cohort of black hypertensive subjects in sub-Saharan Africa.

## METHODS

### Design

It is an observational cross-sectional study.

### Subjects

We prospectively studied every consecutive subject >18 years who were referred for the first time to the Cardiology Clinic of the University of Abuja Teaching Hospital with HT as the primary reason for referral from April 2006 to April 2013. These patients were typically referred by General and Family Physicians in Primary and Secondary Healthcare facilities including private set-ups. Subjects were on average public servants, traders, businessmen, politicians, farmers, artisans, and retired public servants. A total of 2398 subjects were recruited for the study with an average recruitment of 29 subjects per month. Twenty subjects (0.83%) were excluded from the study because of incomplete data at recruitment. Subjects were classified into 3 groups after echocardiography: those with HT and heart failure (HF) abbreviated as HHF, those with HT and left ventricular hypertrophy (LVH) abbreviated as HTLVH, and those with HT without HF and LVH abbreviated as HT. The study was approved by the University of Abuja Teaching Hospital Ethics Clearance Committee.

### Study Data

Standardized questionnaires were used to collect baseline demographic and clinical characteristics of patients. In order to keep selection bias to a minimum, data were collected on a consecutive patient basis. Information obtained included the patients’ age, sex, history of HT, history of diabetes mellitus, and family history of HT and diabetes mellitus. Other information collected was past or present history of myocardial infarction, angina pectoris, cerebrovascular accident, HF and renal failure.

Each subject had a full physical examination including height and weight measurements. Blood pressure measurement was according to standard guidelines with a mercury sphygmomanometer (Accosson, London, UK). Systolic and diastolic blood pressures were measured using Korotkof sounds I and V. Blood pressure was measured at the right arm 3 times, with a 5 minute rest between each measurement. The diagnosis of HT was made when the average of 3 blood pressure measurements was ≥140/90 mm Hg or the subject was already on antihypertensive therapy.^7^ The diagnosis of HF was made according to the recommendation of the European Society of Cardiology.^8^ Ischemic heart disease was diagnosed on the basis of clinical history (presence of chest pain) electrocardiography and cardiac Troponin I >0.5 ng/mL. Subjects were asked to fast for 8 to 12 hours before blood sample collection. Blood chemical analysis was performed at a certified central laboratory. Blood fasting glucose and lipids were analyzed enzymatically by autoanalyzer (Erber spectrophotometer).

### Echocardiography and Electrocardiography

Echocardiography was performed using a commercially available ultrasound system (Vivid E). Subjects were examined in the left lateral decubitus position using standard parasternal, short-axis, and apical views. Studies were performed according to the recommendations of the American Society of Echocardiography^9^ by experienced echocardiographers. Measurements were averaged >3 cardiac cycles. The LV measurements taken include interventricular septal thickness at end diastole (IVSTd), the posterior wall thickness at end diastole (PWTd), the left ventricular (LV) end diastolic diameter (EDD), and LV end systolic diameter. LV systolic function was calculated by Teichholz formula.^10^ Left ventricular mass (LVM) was calculated using the formula^11^: LVM = 0.8 [1.04 (IVSTd + LV EDD + PWTd d) 3 + 0.6 g].

Relative wall thickness was calculated as 2 × posterior wall thickness/LV internal dimension in diastole. LVM was considered to be increased when LVM index (LVMI) exceeded 49.2 g/m^2^^7^ in men and 46.7 g/m^2^^7^ in women.^12^ LV inflow velocities were measured using pulsed-wave Doppler from the apical 4-chamber view with the sample volume located between the tips of the mitral valve leaflet during ventricular diastole. Peak velocity of early rapid filling (E), peak velocity of late filling caused by atrial contraction (A), and the interval from peak of E wave to its extrapolation to the baseline or deceleration time (DT) were measured. The ratio of peak E-wave to A-wave was calculated. Diastolic function was categorized using mitral inflow parameters. Grade 3 diastolic dysfunction or restrictive filling pattern was defined as E/A ratio >2 with DT <130 ms. Grade 1 diastolic dysfunction was defined as E/A ratio <1 and DT >220 ms, whereas grade 2 diastolic dysfunction or pseudo normal filling was diagnosed when DT was >220 ms and the E/A ratio was between 1 and 2 and with the use of Tissue Doppler Imaging.

Right ventricular (RV) systolic function was assessed on echocardiography using M-mode recordings through the lateral tricuspid valve annulus for the purpose of measuring the tricuspid annular plane systolic excursion (TAPSE). TAPSE is a method to measure the distance of systolic excursion of the RV annular segment along its longitudinal plane, from a standard apical 4-chamber window. TAPSE represents longitudinal function of the right ventricle. It is inferred that the greater the descent of the base in systole, the better the RV systolic function. TAPSE is acquired by placing an M-mode cursor through the tricuspid annulus and measuring the amount of longitudinal motion of the annulus at peak systole.^13^ 12-lead electrocardiogram (ECG) was performed for each subject by 2 trained ECG technicians with subsequent independent coding according to Minnesota criteria.^14^

### Statistical Analysis

SPSS software version 16.0 (SPSS Inc, Chicago, IL) was used for statistical analysis. Continuous variables were expressed as mean ± SD. One-way ANOVA with Sheffe post hoc test was used for the comparison of demographic, clinical, laboratory, and echocardiographic parameters among the different group of subjects.

## RESULTS

### Demographic and Clinical Characteristics of Subjects by Sex

**FIGURE 1:**
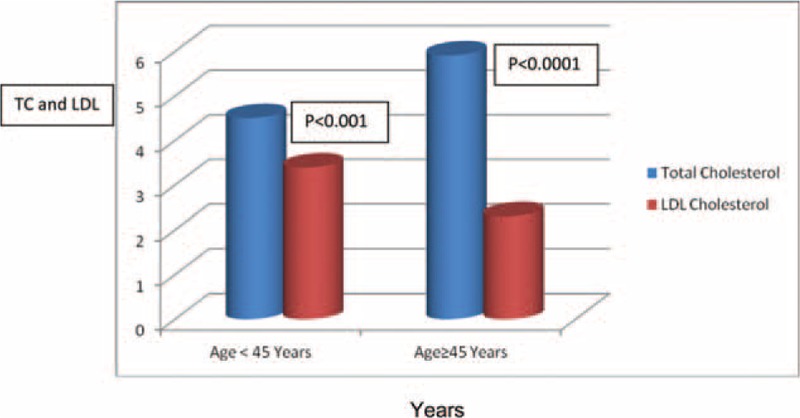
Fasting levels of total cholesterol and LDL cholesterol by age adjusted for sex. LDL = low-density lipoprotein, TC = total cholesterol.

**FIGURE 2:**
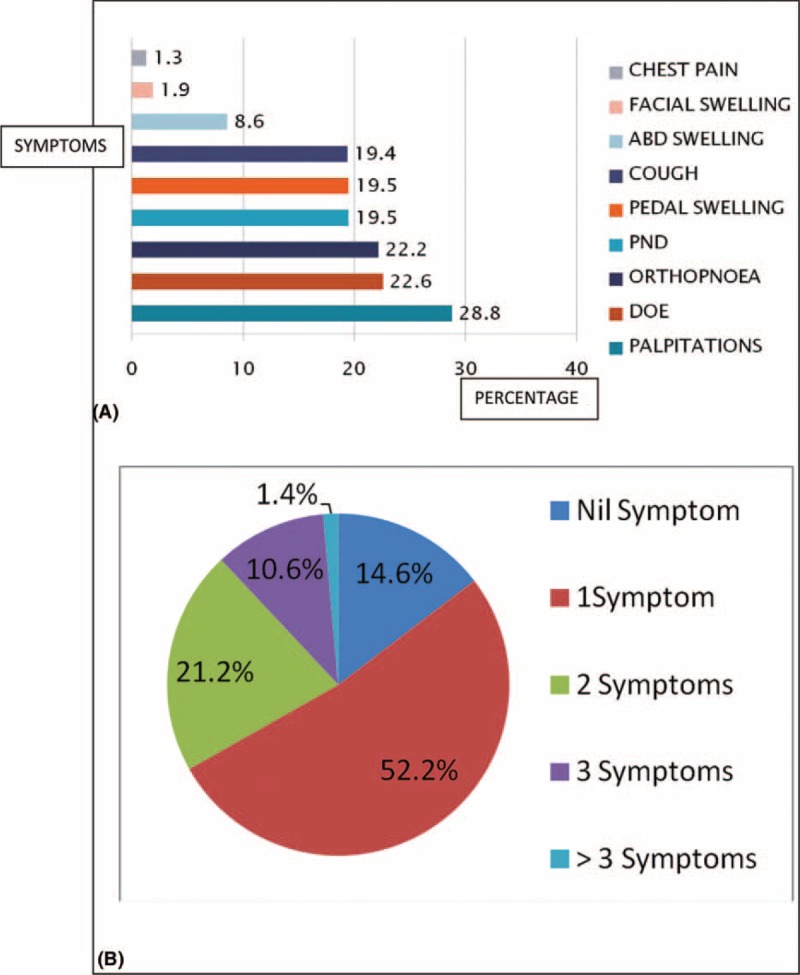
(A) Clinical presentation of subjects in percentage. (B) Pattern of the number of symptoms presented by the subjects. DOE = dyspnoea on exertion, PND = paroxysmal nocturnal dyspnoea.

As shown in Table 1, female subjects had higher body mass index (BMI), total cholesterol and low-density lipoprotein (LDL) cholesterol, and lower packed cell volume when compared with the male subjects. Also, subjects who are 45 years had worse lipid profile compared with their younger counterparts as shown in Figure 1. The male subjects presented more with complications like cerebrovascular accident, chronic kidney disease, LVH and HF when compared with the female subjects as shown in Table 1. Palpitation was the single commonest presentation and commoner than dyspnoea on exertion (28.8% vs 22.6%, *P* < 0.0001) as shown in Figure 2A.

**TABLE 1:**
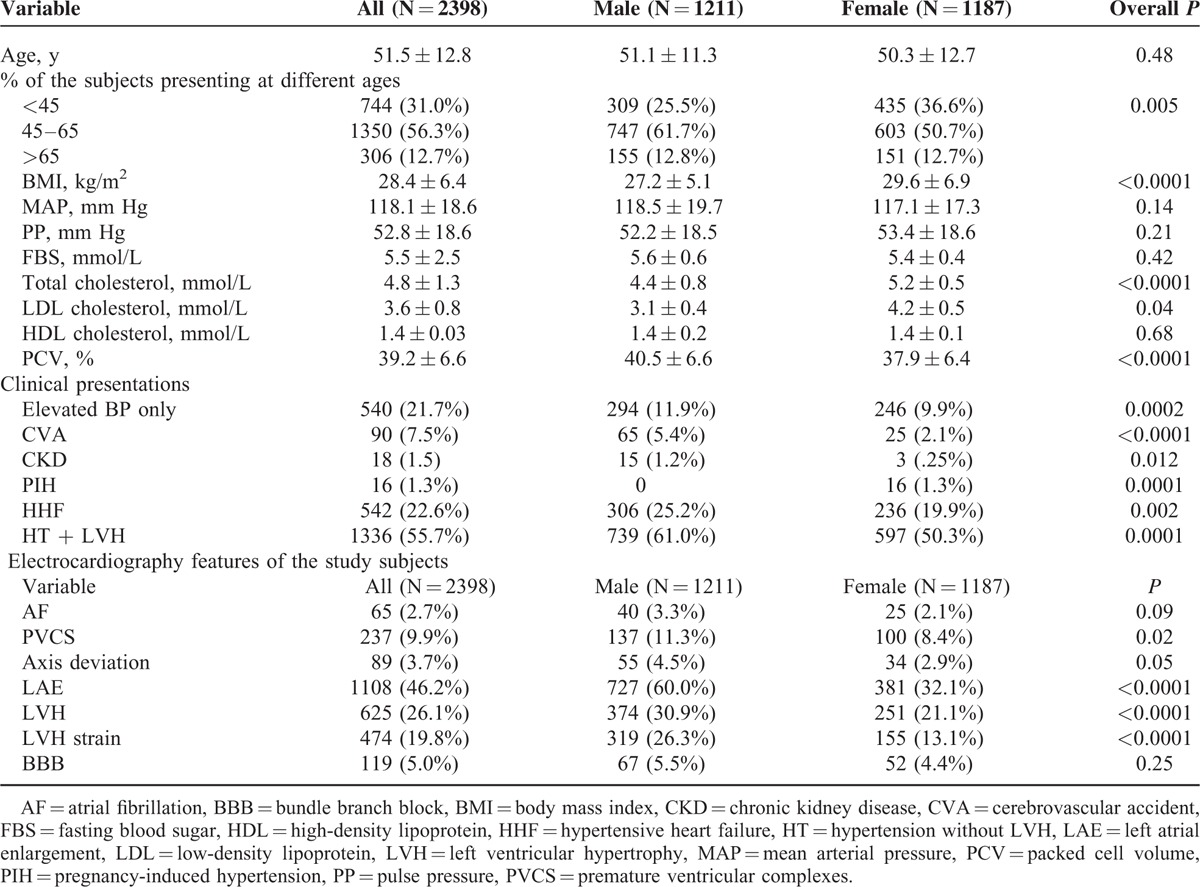
Demographic, Clinical, and Electrocardiography Characteristics of the Subjects

Fifty-two percent of the subjects presented with >1 symptom, whereas the remaining 47.8% presented with 2 or more symptoms as shown in Figure 2B.

**FIGURE 3:**
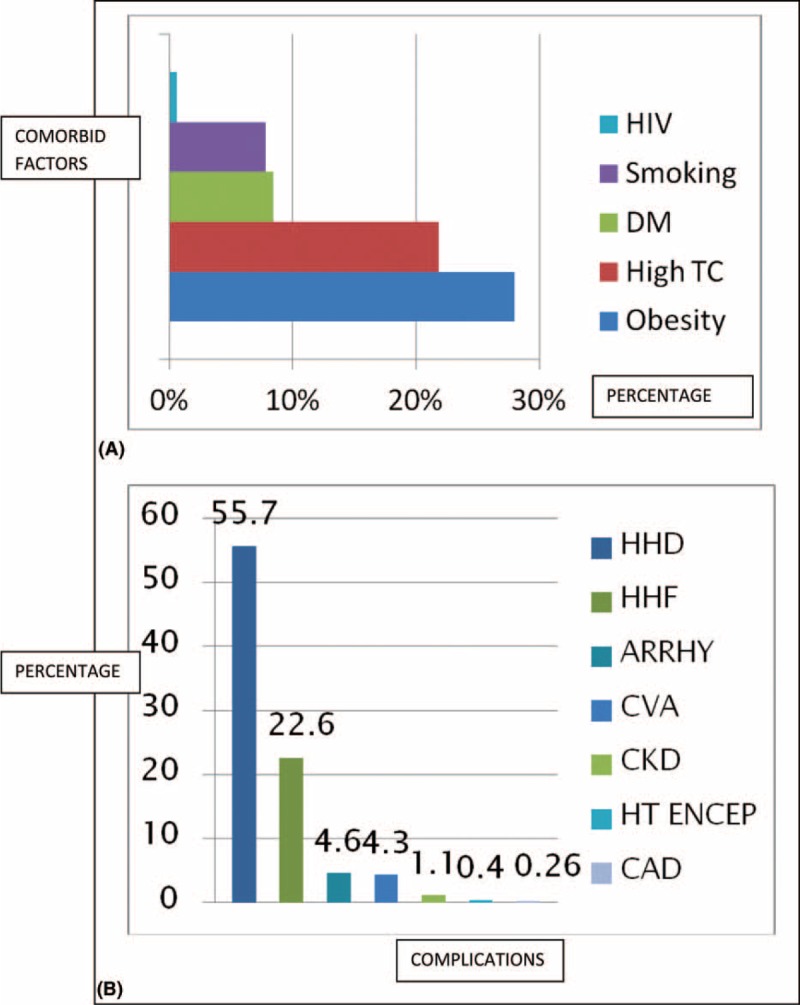
(A) Comorbid factors in the study cohort. (B) Pattern of distribution of complications in the subjects. ARRHY = arrhythmia, CAD = coronary artery disease, CKD = chronic kidney disease, CVA = cerebrovascular accident, DM = diabetes mellitus, Encep = encephalopathy, HHD = hypertensive heart disease, HHF = hypertensive heart failure, HT = hypertension, TC = total cholesterol.

Obesity was the commonest comorbid factor in this cohort followed by hypercholesterolaemia, whereas concomitant retroviral disease occurred in 0.6% of cases as shown in Figure 3A. LVH was the commonest complication in this cohort and significantly higher than HF (55.7% vs 22.6%, *P* < 0.0001) and HF were present in >40% of subjects as shown in Figure 3B.

### Demographic and Clinical Profile of the 3 Main Subjects Groups

Table 2 shows that subjects with HT and left ventricular hypertrophy (HTLVH) and those with hypertensive heart failure (HHF) constitute 78.3% of the total 2398 subject population, and patients with HT with no LVH or HF (HT) were the youngest, HHF subjects were the oldest. Subjects with HTLVH had the highest levels of mean arterial blood pressure and pulse pressure among the cohort, whereas subjects with HHF had the lowest levels of both pulse pressure and mean arterial pressure. Subjects with HHF had the lowest BMI, whereas those with HT had the highest BMI (*P* < 0.0001). There was no statistically significant difference among the study populations in the levels of fasting blood sugar, urea, creatinine, and hemoglobin concentration. The total cholesterol was, however, highest in the HT group and significantly higher than the HHF group (*P* = 0.04).

**TABLE 2:**
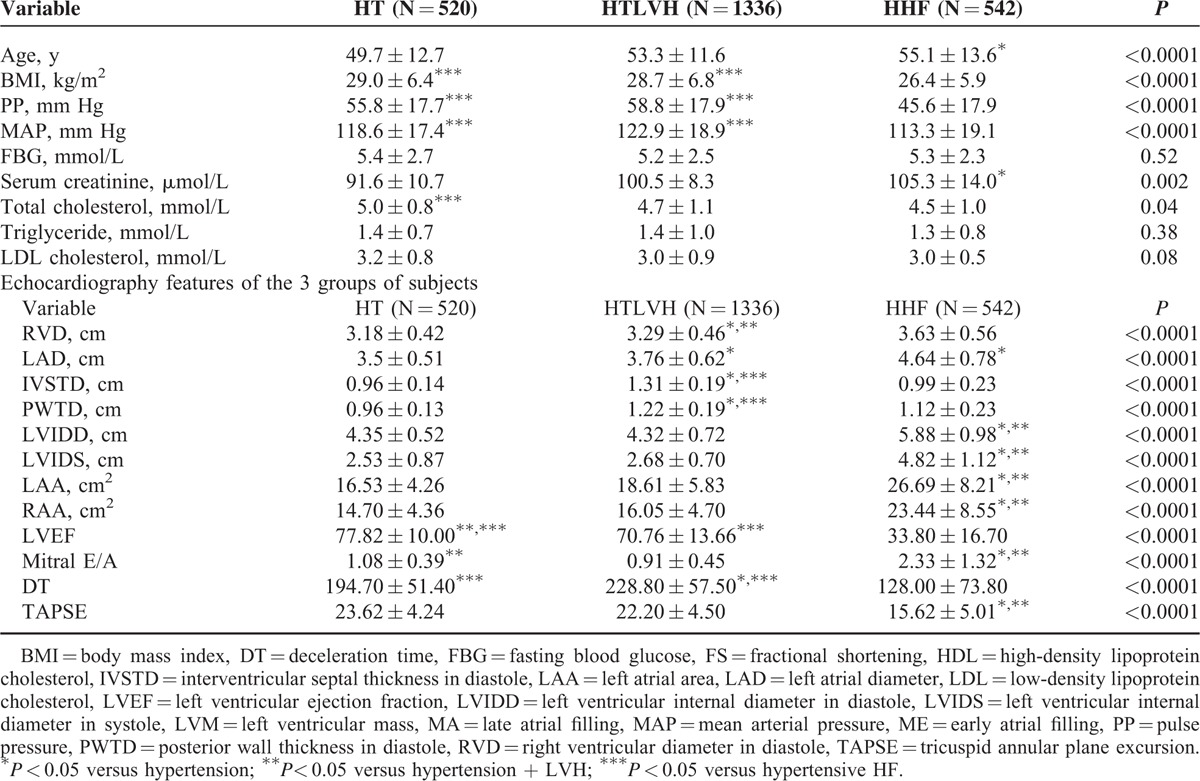
Demographic, Clinical, and Echocardiography Features in the 3 Study Groups

### Electrocardiography Profile in Male and Female Subjects

Table 1 shows the electrocardiography features of the subjects. The male subjects had higher premature ventricular complexes, left atrial enlargement, and LVH with or without strain on electrocardiography. The female subjects, however, had more electrocardiography in normal sinus rhythm compared with the male subjects (*P* < 0.0001).

### Echocardiographic Findings in the 3 Groups of Subjects

The echocardiographic characteristics of the 3 study cohorts are shown in Table 2. As expected, subjects with HTLVH had significantly greater interventricular and LV posterior wall thickness when compared with HT and HHF subjects. They also had higher LVM and LVMI when compared with HT subjects, but had a smaller LVM when compared with HHF subjects. Subjects with HT had significantly higher LVEF when compared with HTLVH and HHF subjects. The largest chamber diameters apart from the right atrial area were seen in HHF. They also had the highest mitral “E”/“A” ratio and the lowest TAPSE value.

### Univariate and Multivariate Regression Analyses Demonstrating Factors that Correlate With Blood Pressure in the Study Population

Tables 3–5,, show factors that correlate with systolic blood pressure, diastolic blood pressure, and mean arterial blood pressure, respectively. The model using diastolic blood pressure has both the highest coefficient of multiple determinations (*R*^2^) and *P* value.

**TABLE 3:**
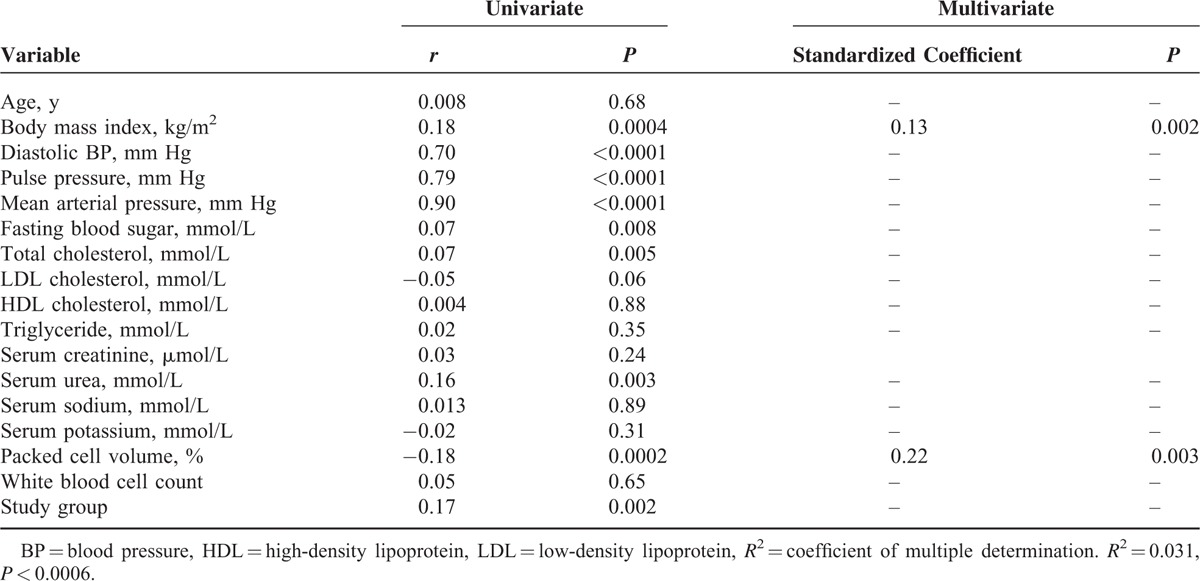
Univariate and Multivariate Regression Analysis Demonstrating Factors That Correlate With Systolic Blood Pressure in the Study Population

**TABLE 4:**
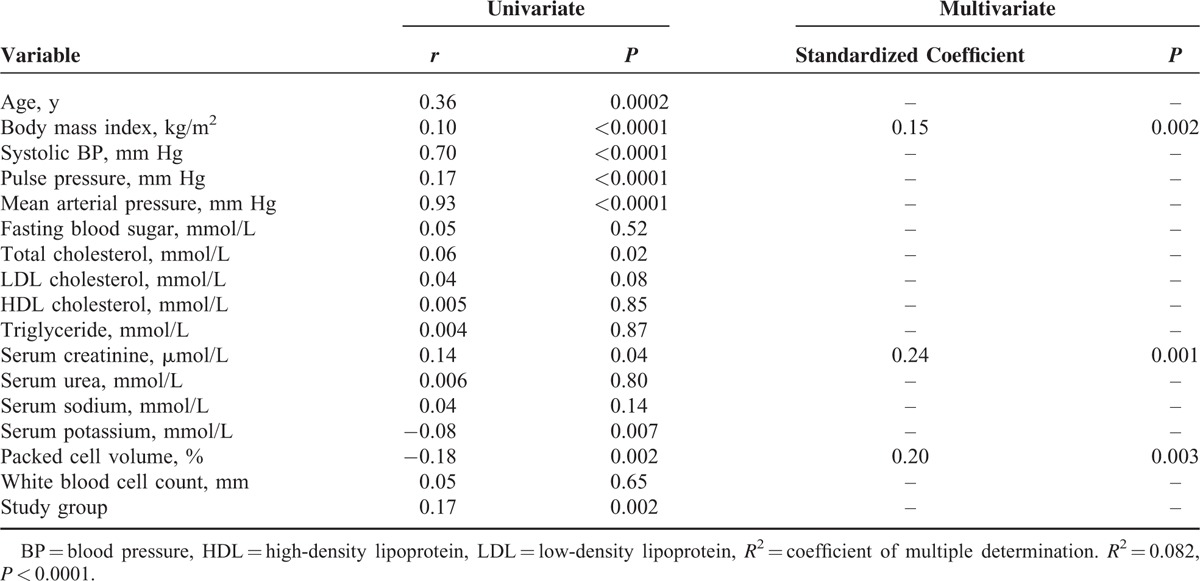
Univariate and Multivariate Regression Analysis Demonstrating Factors That Correlate With Diastolic Blood Pressure in the Study Population

**TABLE 5:**
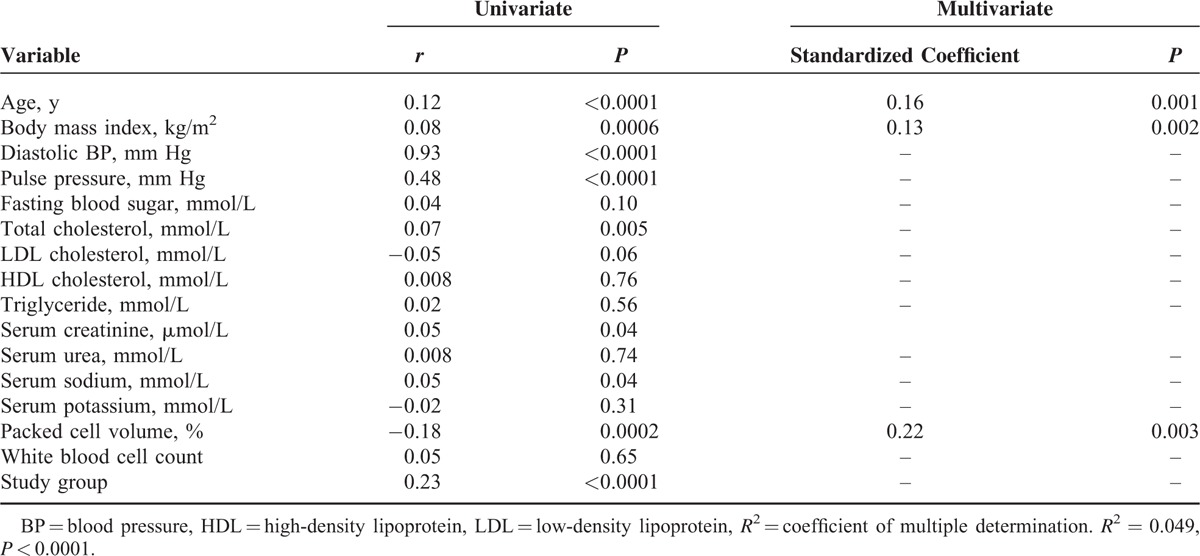
Univariate and Multivariate Regression Analysis Demonstrating Factors That Correlate With Mean Areterial Blood Pressure in the Study Population

## DISCUSSION

In this cohort of black hypertensive subjects referred to a large teaching hospital with a primary diagnosis of HT, we observed that HF was already present in almost 1 in 4 cases. As HT has been previously described as a dominant cause of HF in Black subjects, this is not a surprising finding.^6,15^ In addition, HHF is the commonest form of HF in this population.^16,17^ LV hypertrophy as detected using echocardiography was present in about 56% of cases, as compared with 40% in the Heart of Soweto Study.^18^ The higher prevalence of LVH in the Abuja study supports previous findings of higher prevalence of hypertensive LVH in Blacks compared with Caucasians;^19,20^ whereas the Soweto study was a mixed population of Blacks and Caucasians, the Abuja study only involved Blacks. Although LVH was defined in the Heart of Soweto Study as interventricular septal wall thickness in diastole and or LV posterior wall thickness in diastole >13 mm, we estimated LVH in this study using LVMI.

The proportion of our female subjects presenting at the young age (<45 years) is more compared with the male subjects. This may be due to the fact that many women have their HT discovered at an earlier age compared with the men because of the benefit of having it checked during antenatal visits may account for this finding. The earlier presentation by our women might be partly responsible for the lesser complications like cerebrovascular accident, chronic kidney disease, and HF compared with the male cohort. Supporting previous reports,^21^ a higher proportion of the male subjects presented more at the middle age category (45–65 years) compared with the female subjects, and this is attributed to higher complications of HT like cerebrovascular accident, chronic kidney disease, and HF occurring more at this age compared with female subjects. Presentation at this age will undermine national productivity as a consequence of the number of active life years lost by the most active and experienced workforce of the population. The need, therefore, for the national and regional governments to incorporate mechanisms for routine medical checkup and subsequent early detection of risk factors for cardiovascular disease cannot be overemphasized.

Considering the low prevalence of clinically detected coronary artery disease in this cohort, the high prevalence of LVH and HF in our cohort may further support the theory of progression of HT through hypertensive LVH to HHF without ischemic heart disease.^22^ Although the mechanism of such progression from concentric LVH to HF has not been well defined in hypertensive Black subjects, it has been shown that about 1 in 5 of hypertensive African-Americans with LVH develop impaired systolic function after a medium follow-up period of 4 years.^23^

Palpitation was the single most common reason for initial clinical presentation in this cohort similar to the findings in the Heart of Soweto study,^18^ and may be attributed to structural changes in the heart. Hypertensive encephalopathy which is an acute organic brain syndrome resulting from severely blood pressure was seen in 0.4% of our cohort, further illustrating the burden of HT in our population group.

Our subjects with HHF were about 2 decades younger than subjects with HF in Western Europe and the United States.^24^ This younger age of presentation will undermine national productivity as a consequence of the number of active live years lost by the most active workforce of the population. This finding confirms the critical importance of early detection and treatment of HT in this population group. Fortunately, HT can be readily controlled with medications such as thiazides and calcium channel antagonists which are both affordable and accessible.

Hypercholesterolemia was found in about 22% of our subjects supporting the epidemiological transition in disease pattern in most parts of sub-Saharan Africa.^25^ This is particularly worrisome when one compares this with the Non-Communicable Disease Survey in Nigeria conducted in 1997, which identified hypercholesterolemia in only 8.2% of the 4.3 million hypertensive subjects studied.^26^ This type of change which is noticeable in most low- and middle-income countries can be attributed to dramatic changes occurring in the diet of these populations and has been referred to as the nutrition transition.^27^ On the contrary, increasing urbanization and the trends that accompany it like higher incomes, exposure to mass media and marketing campaigns, greater female employment, and less leisure time are some of the social factors attributed for these changes in diet seen in low- and middle-income countries like Nigeria.^28^ Therefore, with the more recent increase in the gross development product of Nigeria rising to the largest economy in Africa, there is likely going to a further increase in the consumption of these processed foods, thereby leading further to increased prevalence of obesity, dyslipidemia, diabetes mellitus, and the consequent cardiovascular sequel.

Although the prevalence of diabetes mellitus of 8.4% and smoking of 7.8% is much lower than those reported in Europe and the United States, this cannot not be overlooked when one considers the overall lifelong disease burden imposed on the background of HT in young adults.

The female subjects had higher BMI, total cholesterol, and LDL cholesterol when compared with the male subjects in keeping with previous findings in African-Americans.^29^ Also, supporting previous findings we found that middle-age male and female subjects had worse lipid profile compared with the younger subjects as shown in Figure 1, which may partly explain the higher incidences of cerebrovascular accident and coronary artery disease in this age group. It is well known that dyslipidaemia is risk factor for cerebrovacular accident and coronary artery disease.^30^ The difference in lipid between young women and middle-age women has been attributable to hormonal changes.^31^

The male subjects smoke more than the female subjects, which may partly account for the higher prevalence of coronary artery disease among male subjects compared with female subjects. The prevalence of smoking of 7.8% is much lower than that reported in similar subjects in the Heart of Soweto study,^6^ which may partly account for the lower prevalence of ischemic heart disease.

## LIMITATION

Despite adhering to Strobe guidelines^32^ in this study, there are some limitations. First, because the diagnosis of ischemic heart disease myocardial infarction was made with electrocardiography and cardiac enzymes, with no myocardial perfusion scanning or coronary angiography performed, there might have been an under estimation of the prevalence of coronary artery disease in this cohort. But it could be justified due to the low prevalence of ischemic heart disease in Nigeria that routine coronary angiography and myocardial perfusion scanning for asymptomatic subjects are not indicated. However, the careful and extensive phenotyping of our study population with respect to clinical, echocardiographic, and biochemical parameters must have reduced the effect of these limitations.
